# Hyperacute Iatrogenic Atrial Septal Defect Expansion Driven by Tricuspid Regurgitation Jet Impingement

**DOI:** 10.1016/j.jaccas.2026.108009

**Published:** 2026-04-24

**Authors:** Yasuyo Takeuchi, Mizuhiko Ishigaki, Sung-Hae Kim, Norie Mitsushita, Hiroki Sakamoto

**Affiliations:** a Department of Cardiology, Shizuoka General Hospital, Shizuoka, Japan; b Department of Cardiology, Shizuoka Children's Hospital, Shizuoka, Japan

**Keywords:** atrial septal defect closure, cardiogenic shock, iatrogenic atrial septal defect (iASD), mitral transcatheter edge-to-edge repair (M-TEER), tricuspid regurgitation (TR)

## Abstract

**Background:**

Iatrogenic atrial septal defects (iASDs) after mitral transcatheter edge-to-edge repair (M-TEER) are generally benign. However, advanced atrial remodeling can trigger catastrophic mechanical failure via rapid morphologic expansion.

**Case Summary:**

An 84-year-old woman with atrial fibrillation and diastolic dysfunction underwent M-TEER. Despite a stable intraprocedural iASD, she developed cardiogenic shock within 3 hours. Three-dimensional transesophageal echocardiography documented hyperacute doubling of the defect area. The mechanism was direct tricuspid regurgitation (TR)-jet impingement on the thinned septum, reflecting severely impaired left atrial compliance. Emergency iASD closure using “stationary deployment” prevented further traumatic tearing, resulting in immediate hemodynamic stabilization.

**Discussion:**

This case identifies TR-jet impingement as a predictor of acute iASD expansion. Transesophageal echocardiography is essential for monitoring this mechanical vicious cycle.

**Take-Home Messages:**

Impaired atrial compliance and TR-jet impingement can cause rapid iASD enlargement after M-TEER. Prompt recognition and closure are vital for managing this life-threatening complication.

## Visual Summary

**Figure:**
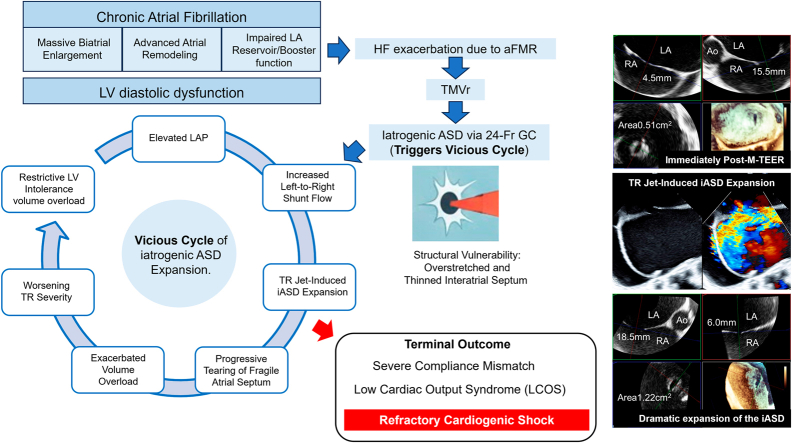

## Past Medical History

An 84-year-old woman with hypertension and long-standing atrial fibrillation (AF) presented with worsening dyspnea. Despite intensive medical therapy, her clinical status remained refractory.

**Take-Home Messages**

• Tricuspid regurgitation jet as a mechanical driver: direct tricuspid regurgitation jet impingement on an iatrogenic atrial septal defect can act as a “hydrodynamic wedge,” triggering hyperacute expansion in remodeled, friable atria.
• The “stationary deployment” strategy: for extremely friable septal rims, a meticulous imaging-guided “stationary deployment” technique—avoiding balloon sizing and the “wiggle test”—prevents further traumatic tearing during defect closure.
• The role of advanced atrial cardiomyopathy: clinicians should recognize that a “burnt-out” left atrium with poor compliance is highly vulnerable to acute pressure changes, potentially turning a standard iatrogenic atrial septal defect into a life-threatening complication.

## History of Presentation

On admission for congestive heart failure, coronary angiography showed no obstruction. Right heart catheterization revealed elevated filling pressures: mean right atrial pressure 13 mm Hg, mean pulmonary capillary wedge pressure 23 mm Hg, and pulmonary artery pressure 40/21 mm Hg (mean: 28 mm Hg) (Table 1).

**Table 1:** Clinical Course and Hemodynamic Timeline

Timeline	Events
Date of submission/day 1	An 84-year-old woman presented with NYHA functional class Ⅳ symptoms and decompensated systolic congestive heart failure requiring emergency department admission. ECG showed atrial fibrillation. Echo showed severe mitral regurgitation and moderate tricuspid regurgitation.
Day 8	She underwent intravenous diuresis and clinically improved, but severe MR due to a flat mitral valve with coaptation gap was seen.
Day 9	Heart Team discussion: the patient was deemed high risk for surgical mitral valve repair or replacement, and transcatheter edge-to-edge repair (TEER) was considered.
Day 15	The patient underwent a complex mitral TEER procedure with mild residual MR. Postprocedure observation: sudden hypotension, tachycardia, and respiratory distress. Echo showed mild MR, but severe tricuspid regurgitation with IVS flattening (D-shape), and iASD with significant shunt. Emergency RHC: confirmed low-output syndrome (CI 1.44 L/min/m^2^) and significant left-to-right shunt (Q_p_/Q_s,_ 2.09).
Day 15	Emergency iASD closure: successful placement of an 18-mm Occlutech device.
Day 17	Extubated and hemodynamics stabilized.
Day 37	Thirty-day clinical follow-up showed that the patient improved to NYHA functional class Ⅱ, and Echo showed mild MR.
Day 39	The patient was discharged to home.

A comprehensive chronological mapping of the case, from initial presentation with severe atrial functional mitral regurgitation to the 1-month follow-up. It highlights the critical 3-hour window during which hemodynamic collapse ensued, directly correlating with the rapid, mechanical expansion of the iASD.

CI = cardiac index; ECG = electrocardiogram; Echo = echocardiography; iASD = iatrogenic atrial septal defect; IVS = interventricular septum; MR = mitral regurgitation; Q_p_/Q_s_ = pulmonary-to-systemic flow ratio; RHC = right heart catheterization.

## Initial Investigations

**Figure 1:**
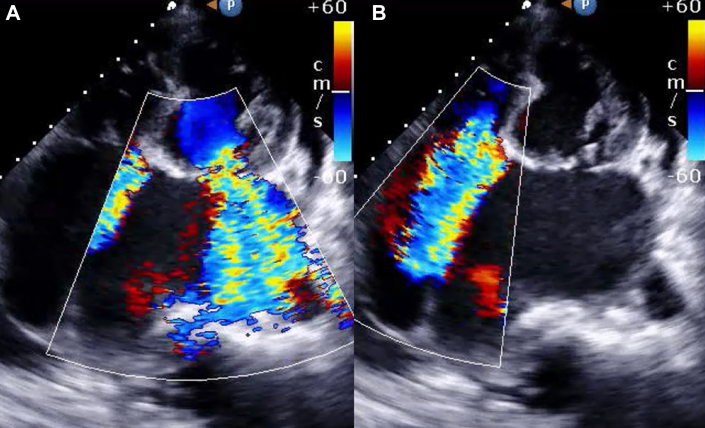
Transthoracic Echocardiography Upon Admission Apical 4-chamber view with color Doppler. (A) Severe atrial functional mitral regurgitation with a significant eccentric jet. (B) Moderate-to-severe functional tricuspid regurgitation. The massive biatrial enlargement and advanced remodeling serve as the mechanical substrate for subsequent hemodynamic deterioration.

Initial transthoracic echocardiography demonstrated severe atrial functional mitral regurgitation (MR) with a “flat” mitral valve morphology and moderate-to-severe tricuspid regurgitation (TR) (Video 1, Figures 1A and 1B). Although medical optimization partially alleviated the TR, severe MR persisted. Given high surgical risk (society of thoracic surgeons score: 12.0%), the Heart Team performed mitral transcatheter edge-to-edge repair (M-TEER).^1^

## Differential Diagnosis

The differential diagnosis for postprocedural hemodynamic collapse included:

1. Acute cardiac tamponade: immediately excluded by emergency echocardiography.
2. Worsening right heart failure: potentially exacerbated by the large 24-F guiding catheter's impact on the tricuspid valve or right ventricle function.
3. Recurrent MR or single-leaflet device attachment (SLDA): SLDA or clip instability causing MR recurrence was considered; however, transesophageal echocardiography (TEE) confirmed a well-seated clip and durable MR reduction.
4. Acute iatrogenic atrial septal defect (iASD) disruption: identified through serial 3-dimensional (3D) TEE, which revealed a hyperacute increase in the shunt area.

## Investigations and Clinical Course

**Figure 2:**
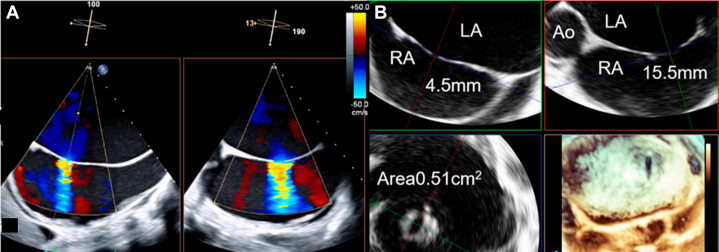
Baseline iASD Assessment Immediately Post-M-TEER Intraprocedural transesophageal echocardiography at the conclusion of the procedure. (A) Two-dimensional (2D) color Doppler showing the initial left-to-right shunt after 24-F sheath withdrawal. (B) 3D quantification analysis demonstrating an iatrogenic atrial septal defect (iASD) orifice area of 0.51 cm^2^ (major/minor axis: 15.5/4.5 mm). LA = left atrium; M-TEER = mitral transcatheter edge-to-edge repair; RA = right atrium.

TEER was performed using a fourth-generation system. A single MitraClip G4 NTW (Abbott Vascular) was selected and deployed at the A2/P2 position. This clip size ensured broad grasping and stable coaptation for “flat” morphology while minimizing mechanical stress. After clip deployment, 3D quantitative analysis confirmed a stable baseline iASD: major axis 15.5 mm, minor axis 4.5 mm, and area 0.51 cm^2^ (Video 2, Figures 2A and 2B).

**Figure 3:**
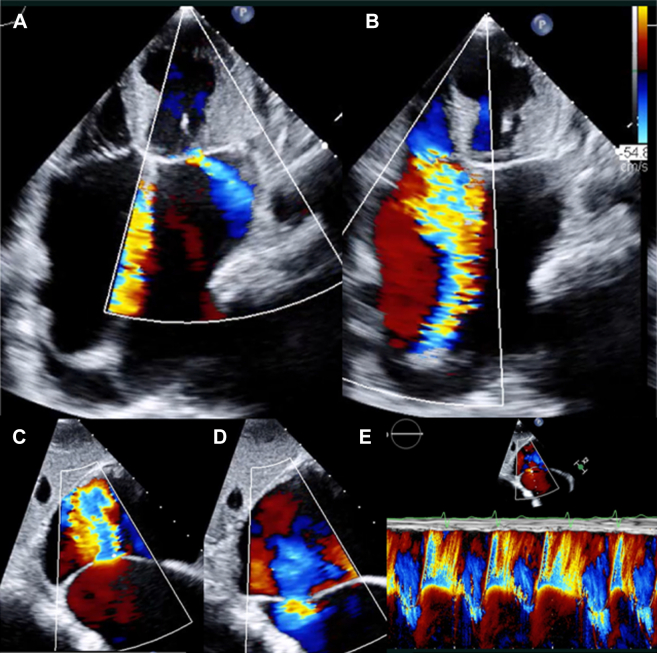
Acute Hemodynamic Collapse and Mechanical Ominous Sign Bedside transthoracic echocardiography during cardiogenic shock. (A) Successfully reduced mitral regurgitation (mild) but significantly exacerbated severe tricuspid regurgitation (TR). (B) High-velocity TR jet directly impinging on the interatrial septum. (C and D) Subcostal view revealing a large iatrogenic atrial septal defect with bidirectional shunting: (C) left-to-right and (D) right-to-left flow. (E) Color Doppler M-mode confirming the complex bidirectional shunt.

Immediately post-procedure, the patient developed persistent AF with rapid ventricular response. Although initially stable, this tachycardia gradually worsened, culminating in refractory hypotension and respiratory distress 3 hours post-procedure. Urgent TEE revealed preserved left ventricular (LV) function (ejection fraction 45%-52%) and successful MR control (grade I, mean pressure gradient, 4 mm Hg). In striking contrast, right-sided failure had progressed rapidly: the right atrium was severely dilated, TR worsened to severe, and estimated systolic pulmonary artery pressure rose to 66 mm Hg (Figures 3A and 3B). Subcostal views and color M-mode confirmed a large iASD with a complex bidirectional shunt (Figures 3C to 3E).

**Figure 4:**
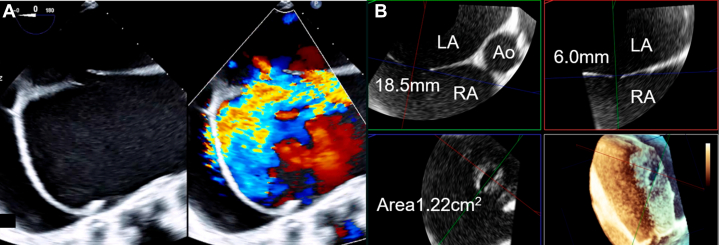
Rapid Morphological Evolution Emergency transesophageal echocardiography during the acute phase. (A) Side-by-side 2-dimensional B-mode and color Doppler views capturing the high-velocity tricuspid regurgitation jet surging directly into the iASD, acting as a “hydrodynamic wedge.” (B) Repeat 3-dimensional quantification analysis documenting a dramatic expansion of the iASD; the orifice area more than doubled to 1.22 cm^2^ (major/minor axis: 18.5/6.0 mm). This rapid evolution underscores the mechanical fragility of the remodeled septum under the mechanical stress of high-kinetic eccentric jet impingement. iASD = iatrogenic atrial septal defect; LA = left atrium; RA = right atrium.

Repeat 3D quantitative TEE showed dramatic iASD expansion. Three-dimensional color Doppler imaging captured the high-velocity TR jet surging directly into the iASD, acting as a “hydrodynamic wedge” (Video 3, Figure 4A). Quantitative analysis confirmed this rapid morphologic evolution, with the orifice area more than doubling to 1.22 cm^2^ (Figure 4B). Hemodynamic stability was not restored despite catecholamine support. Right heart catheterization confirmed a severely depressed cardiac index (1.44 L/min/m^2^) and a significant oxygen step-up (Q_p_/Q_s_ 2.09), identifying iASD disruption as the primary driver of shock (Table 2). Furthermore, the subsequent 10% decline in left ventricular ejection fraction post-closure reflects an acute afterload mismatch, unmasking latent ventricular cardiomyopathy^1^ by eliminating the remaining right-to-left shunt.

**Table 2:** Longitudinal Changes in Echocardiographic and Hemodynamic Parameters

	Baseline (Admission)	Baseline (Pre-M-TEER)	Post-M-TEER (Pre-iASD Closure)	Post-Closure (POD 37)
Echocardiographic findings				
MR severity (grade)	Severe	Severe	Mild	Mild-moderate
MR volume (PISA, mL)	79	63	14	21
MR EROA (PISA, cm^2^)	0.45	0.35	0.17	0.16
TR severity (grade)	Moderate-severe	Mild	Severe-massive	Moderate
Jet area/central RA area (%)	45.6	24.5	46.6	34.6
TR vena contracta (mm)	6.7	2.9	14.1	4.7
TR Vmax (m/s)	2.6	2.6	3.6	2.7
LVEF (%)	60	56	52	42
LAVI (mL/m^2^)	–	442	322	310
Hemodynamic data (RHC/SG)				
mRAP (mm Hg)	13	–	18	–
sPAP (mm Hg)	40	–	43	–
mPAP (mm Hg)	28	–	27	–
mPCWP (mm Hg)	23	-	16	-
PVR (WU)	2.02	-	3.09	-
Cardiac Index (Fick, L/min/m^2^)	1.67	-	1.44	-
Q_p_/Q_s_	1.00	-	2.09	-
SvO_2_ (%)	50.5	-	70.7	-

EROA = effective regurgitant orifice area; iASD = iatrogenic atrial septal defect; LAVI = left atrial volume index; LVEF = left ventricular ejection fraction; M-TEER, mitral transcatheter edge-to-edge repair; mPAP = mean pulmonary artery pressure; mPCWP = mean pulmonary capillary wedge pressure; mRAP = mean right atrial pressure; MR = mitral regurgitation; PISA = proximal isovelocity surface area; POD = postoperative day; PVR = pulmonary vascular resistance; Q_p_/Q_s_ = pulmonary-to-systemic blood flow ratio; RA = right atrial; RHC = right heart catheterization; SG = Swan-Ganz; sPAP = systolic pulmonary artery pressure; SvO_2_ = mixed venous oxygen saturation; TR = tricuspid regurgitation; WU = Wood units.

## Management

In profound cardiogenic shock, emergency transcatheter iASD closure was performed under TEE guidance. The defect was highly eccentric (18.5 mm × 6.0 mm) with a visible tear into the fragile septal tissue. Given the extreme friability of the remodeled rims, we avoided balloon sizing and the “wiggle test” to prevent mechanical propagation.

**Figure 5:**
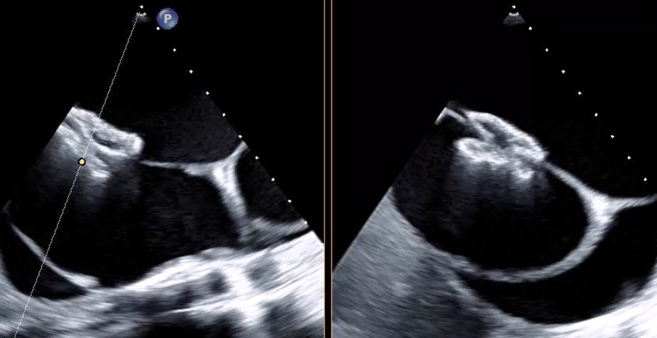
Final Result of Emergency Iatrogenic ASD Closure Transesophageal echocardiography after device release. Still image demonstrating the 18-mm Occlutech Figulla Flex II ASD occluder in its final position. The thinned, overstretched interatrial septum is securely sandwiched between the disks. This was achieved through a stationary deployment technique (Video 4) to avoid further mechanical tearing. The precise alignment ensures complete shunt elimination while preserving the integrity of the friable tissue. ASD = atrial septal defect.

Based on the 18.5-mm major axis, an 18-mm ASD occluder was selected. We used “stationary deployment,” expanding disks in a fixed position with minimal tension (Video 4). After this atraumatic release, the device remained stable, securely sandwiching the overstretched septum (Video 4, Figure 5).

## Follow-Up

**Figure 6:**
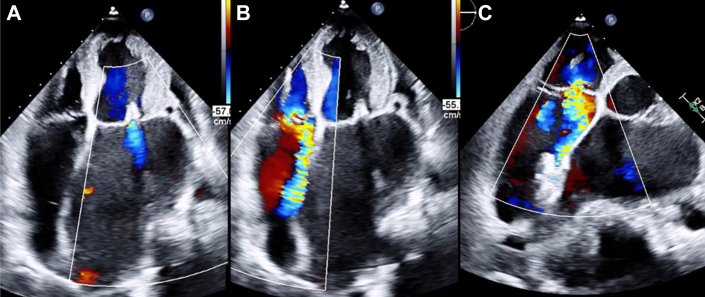
Postprocedural Recovery and Device Stability Transthoracic echocardiography on postprocedural day 1. (A) Stable mitral repair (mild mitral regurgitation) and elimination of the iatrogenic shunt. (B) Reduction in TR to a moderate level. (C) The persistent TR jet is seen directly impinging on the occluder, which acts as a mechanical barrier, protecting the friable septum from further tearing. TR = tricuspid regurgitation.

Immediately after closure, hemodynamics stabilized and catecholamines were tapered within 24 hours. Transthoracic echocardiography on postprocedural day 1 confirmed successful iASD closure with a stable mitral repair showing only mild MR (Figure 6A). Furthermore, previous severe TR improved to a moderate level (Figure 6B). A modified 4-chamber view highlighted that the persistent high-velocity TR jet was now impinging on the occluder, which acted as a mechanical shield for the friable interatrial septum (Video 5, Figure 6C). The patient was discharged on day 39 and remains clinically stable at 3 years.

## Discussion

This case highlights the catastrophic iASD propagation and the need for an imaging-centered, atraumatic closure strategy.^2^^,^^3^

## Mechanistic Insight Into Acute iASD Failure

Although iASD enlargement is typically a chronic process,^3^^,^^4^ this case demonstrates hyperacute expansion within hours. The underlying pathophysiology was rooted in advanced atrial cardiomyopathy^5^ and AF with rapid ventricular response. In patients with chronic LV diastolic dysfunction, the left atrium (LA) initially maintains hemodynamics by modulating its reservoir, conduit, and booster functions.^6^ However, chronic pressure overload eventually exhausts these functional reserves, leading to a “burnt-out” stage where the LA becomes a noncompliant, passively overstretched chamber.

In this patient, stage IV architectural remodeling (European Heart Rhythm Association classification)^5^—marked by an extreme left atrial volume index (442 mL/m^2^)—induced an attenuated, “tissue-paper-like” interatrial septum. Although typical post-TEER iASDs are <10 mm, the initial 15.5-mm major axis in this case resulted from this extreme septal frailty and 24-F sheath manipulation. The defect was highly eccentric and slit-like, with a baseline area of only 0.51 cm^2^, rarely needing immediate closure.^3^^,^^7^

Furthermore, the acute reduction of severe MR eliminated the low-pressure “vent,” triggering an acute increase in LV afterload. The baseline left ventricular ejection fraction of 60% was likely overestimated due to MR venting; its subsequent decline to 45% to 52% reflects an acute afterload mismatch. This sudden pressure surge, combined with the concentrated kinetic energy of the TR jet,^8^ acted as a “hydrodynamic wedge” on the iASD margins.

After the postprocedural hemodynamic collapse, emergency echocardiography excluded clip-related complications, such as SLDA or valvular perforation. TEE confirmed stable clip positioning with durable MR reduction, thereby identifying the massive right-to-left shunt through the expanded iASD (area 1.22 cm^2^) as the primary driver of shock. Crucially, the initial benign appearance of the iASD under stable postprocedural conditions may be deceptive. The synergy between acute LV afterload mismatch and high-velocity TR jet can rapidly exceed the mechanical threshold of a thinned atrial septum.

## The “Mechanical Vicious Cycle” of Hemodynamics

Our findings captured a lethal feedback loop (Central Illustration): the initial iASD led to right ventricular volume overload, worsening functional TR. This increased TR-jet energy then further expanded the iASD, creating an escalating shunt (Q_p_/Q_s_ 2.09). Critically, the TR jet was oriented precisely toward the interatrial septum. In a noncompliant LA, this kinetic energy could not be dissipated, effectively concentrating the hydraulic force on the iASD margins. Although iASD post-M-TEER is often benign,^3^^,^^7^^,^^8^ our case suggests that in patients with severe baseline TR and a remodeled, dysfunctional LA, early closure should be considered if low cardiac output syndrome emerges.

## Considerations for Concomitant Tricuspid Intervention

Concomitant tricuspid intervention remains controversial.^9^^,^^10^ In this case, although the patient initially presented with moderate-to-severe TR, optimized medical therapy successfully downstaged the severity to mild (vena contracta <3 mm and a small central jet area) before TEER. Based on this response and the expectation that MR reduction would further alleviate left-sided filling pressures, we initially opted for a staged strategy.

However, our experience suggests that in advanced atrial cardiomyopathy, even TR that has been successfully downstaged to “mild” can undergo catastrophic “rebound.” Acute afterload mismatch (Table 2) and subsequent left atrial pressure surge likely triggered the rapid deterioration of TR back to a severe level. This sudden surge in right-sided jet energy, striking a septum already strained by left-sided loading, triggered the hyperacute expansion of the iASD. The staged strategy must be balanced against the risk of such unpredictable and fatal hemodynamic shifts.

## Strategic Bailout

When managing acute iASD failure with friable rims, balloon sizing and the “wiggle test” can be hazardous.^2^ In conventional ASD closure, the device is typically pulled against the septum for stability. However, in our patient, such tension could have acted as a secondary mechanical stressor, triggering further tearing. To mitigate this risk, we used a flexible delivery system to maintain a neutral position during disk expansion (“stationary deployment”). By confirming stability through multiple 3D-TEE planes alone and performing a “soft release,” we successfully sealed the defect without extending the existing tear. This approach minimized axial stress on the superior and inferior rims, which were already thinned and prone to further longitudinal splitting.

## Conclusions

Hyperacute expansion of an iASD can lead to fatal hemodynamic collapse, driven by a “mechanical vicious cycle” where a high-velocity TR jet acts as a hydrodynamic wedge against a remodeled, friable interatrial septum. In patients with advanced atrial cardiomyopathy, clinicians should suspect this phenomenon if rapid deterioration occurs post-M-TEER. An imaging-centered, atraumatic closure strategy—using “stationary deployment” and “soft release” techniques—is essential to stabilize hemodynamics and ensure device stability without further septal injury. Ultimately, this case underscores the vital role of a functioning Heart Team, where collaboration between imaging specialists and interventionalists is essential for catastrophic structural complications.

### Patient Perspective

“I am deeply grateful for the emergency treatment that saved my life. Thanks to the rapid response and the care I received, I have been able to enjoy three more years of life in good health. I feel truly fortunate and am more than happy to share my story if it can help other patients and medical professionals.”

### Ethical Statement

This study complies with the Declaration of Helsinki. Written informed consent was obtained from the patient for the procedure and the publication of this case report, including all associated images and videos.

**Visual Summary:**
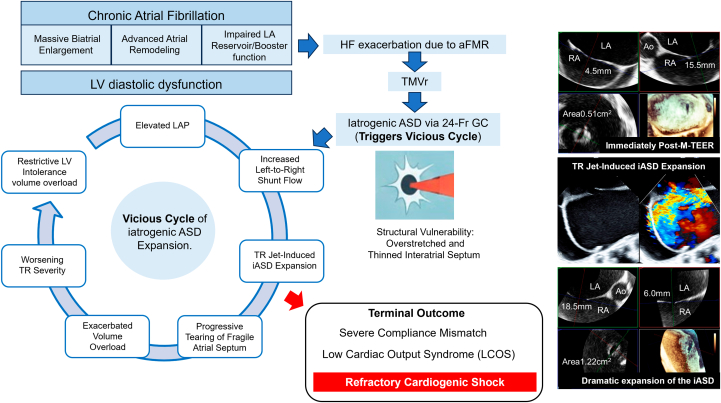
The Mechanical Vicious Cycle of iASD Expansion This figure integrates a mechanistic schema (left) with sequential echocardiographic evidence (right) to illustrate the process of septal failure. Top (Initial State): 3D quantification (3DQ) analysis demonstrates a stable initial iASD with an orifice area of 0.51 cm^2^. Middle (The Trigger): Color Doppler captures the high-velocity TR jet surging directly into the iASD. This illustrates the “hydrodynamic wedge” effect, concentrating mechanical stress on the friable septal margins. Bottom (Catastrophic Expansion): Repeat 3DQ analysis documents a dramatic expansion of the iASD; the orifice area more than doubled to 1.22 cm^2^. Conclusion: In patients with advanced atrial cardiomyopathy, the concentrated kinetic energy of a tricuspid regurgitation jet can act as a “hydrodynamic wedge,” triggering rapid mechanical failure of the atrial septum and subsequent cardiogenic shock. aFMR = atrial functional mitral regurgitation; GC = guiding catheter; HF = heart failure; iASD = iatrogenic atrial septal defect; LA = left atrium; LAP = left atrial pressure; LV = left ventricular; M-TEER = mitral transcatheter edge-to-edge repair; RA = right atrium; TMVr = Transcatheter Mitral Valve Repair; TR = tricuspid regurgitation.

### Declaration of Generative AI and AI-Assisted Technologies in the Writing Process

During the preparation of this work, the authors used Gemini (Google) for English language editing and grammatical corrections. After using this tool, the authors reviewed and edited the content as needed and take full responsibility for the final version.

## Funding Support and Author Disclosures

The authors have reported that they have no relationships relevant to the contents of this paper to disclose.

## Appendix

Video 1 Baseline Valvular Pathologies (Transthoracic Echocardiography) Apical 4-chamber view showing massive biatrial dilatation with severe mitral regurgitation and severe tricuspid regurgitation. The interatrial septum appears highly mobile and thinned, reflecting advanced atrial cardiomyopathy.

Video 2 Immediate Postprocedural Stability Transesophageal echocardiography at the conclusion of mitral transcatheter edge-to-edge repair. Part 1 (biplane 2-dimensional [2D] views) visualizes the longitudinal iatrogenic atrial septal defect corresponding to the 24-F delivery sheath. Part 2 (2D with color Doppler) confirms a purely left-to-right shunt at this stage. Part 3 (3D view without color Doppler) provides a comprehensive en face view of the circumscribed, stable puncture site, without evidence of the mechanical tearing seen later.

Video 3 Mechanical Septal Failure and the “Hydrodynamic Wedge” Mechanism Transesophageal echocardiography performed 3 hours post-procedure during cardiogenic shock. Part 1 (side-by-side 2-dimensional [2D] B-mode and color Doppler views) captures the marked expansion of the iatrogenic atrial septal defect into a large, irregular longitudinal tear. Part 2 (3D en face view) provides a comprehensive visualization of the mechanical “flapping” of the thinned septal rims, confirming a complete mechanical failure of the friable interatrial septum. The concentrated kinetic energy of the rebounded tricuspid regurgitation jet, driven by postprocedural afterload mismatch, acted as a “hydrodynamic wedge” to trigger this rapid propagation of the septal dissection.

Video 4 Emergency Bailout and Final Result The iatrogenic atrial septal defect was successfully closed using an 18-mm Occlutech atrial septal defect occluder. Part 1 (multiplane transesophageal echocardiography) confirms stable positioning of the delivery system. Part 2 (fluoroscopy) demonstrates the “stationary deployment” technique, where the device was carefully deployed without tension to avoid further injury to the friable septal rims. Part 3 (final transesophageal echocardiography) confirms stable device seating, complete resolution of the iatrogenic defect, and immediate hemodynamic stabilization.

Video 5 Postprocedural Stability and Flow Patterns Transthoracic echocardiography performed 1 day after emergency iatrogenic atrial septal defect closure. Part 1 (side-by-side 2-dimensional [2D] B-mode and color Doppler, apical 4-chamber view) visualizes the stable position of the occluder device and mild residual mitral regurgitation. Part 2 (side-by-side 2D B-mode and color Doppler, apical 4-chamber view) reveals massive biatrial dilatation and moderate tricuspid regurgitation. Part 3 (color Doppler) demonstrates the residual tricuspid regurgitation jet directly impinging on the atrial septal occluder, confirming that the device now effectively shields the friable interatrial septum from the kinetic energy of the “hydrodynamic wedge.”
